# ACE- and DPP-IV-Inhibitory Peptides from Bambara Groundnut Hydrolysate: Elucidation Using Computational Tools and Molecular Docking

**DOI:** 10.3390/biology14050511

**Published:** 2025-05-07

**Authors:** Jirakrit Saetang, Thaiyawat Haewphet, Krisana Nilsuwan, Soottawat Benjakul

**Affiliations:** International Center of Excellence in Seafood Science and Innovation, Faculty of Agro-Industry, Prince of Songkla University, Hat Yai 90110, Songkhla, Thailand; jirakrit.s@psu.ac.th (J.S.);

**Keywords:** hydrolysates, metabolic syndrome, legumes, 3D structures

## Abstract

Hypertension and type 2 diabetes are the major metabolic syndromes, often managed using synthetic ACE and DPP-IV inhibitors that may cause adverse effects on health. This study investigated Bambara groundnut protein hydrolysates as a natural source of dual ACE- and DPP-IV-inhibitory peptides. Protein isolates were hydrolyzed using Flavourzyme, and the resulting peptides were fractionated using membranes with different molecular weight cut-offs. Those fractions were then analyzed for enzyme inhibition. Peptides were identified by LC-MS/MS and screened using PeptideRanker and BIOPEP-UWM, followed by molecular docking against ACE (PDB: 1O8A) and DPP-IV (PDB: 1NU6). The >10 kDa and 5–10 kDa fractions showed the highest ACE- and DPP-IV-inhibitory activities, respectively. Some peptides such as YKDGLYSPHW, LPVSTPGKF, and EPWWPK displayed strong binding affinities (ΔG: −10.2 to −11.3 kcal/mol for ACE, −8.6 to −9.1 kcal/mol for DPP-IV) and interacted with key catalytic residues, including His387 and Glu411 in ACE, and Ser630, Glu205, and Phe357 in DPP-IV. These findings highlight the potential of Bambara groundnut hydrolysates or peptides as a source of natural ACE and DPP-IV inhibitors.

## Simple Summary

Hypertension and type 2 diabetes are major global health concerns commonly treated with synthetic drugs that may cause undesirable side effects. In this study, proteins from Bambara groundnut, a resilient and protein-rich legume, were enzymatically broken down to produce bioactive peptides. These peptides were separated by size and evaluated for their ability to inhibit ACE and DPP-IV enzymes: one involved in blood pressure regulation and the other in glucose metabolism. Using advanced techniques including mass spectrometry, computational tools, and molecular docking, several peptides were identified with strong predicted binding and inhibitory effects on both enzymes. These findings highlight Bambara groundnut as a promising natural source of multifunctional peptides that could support the development of functional foods or nutraceuticals aimed at managing metabolic disorders.

## 1. Introduction

The renin–angiotensin system (RAS) plays a crucial role in blood pressure regulation. An enzyme called angiotensin-converting enzyme (ACE) plays a profound role in this system by converting angiotensin I to the potent vasoconstrictor angiotensin II. ACE also inactivates the vasodilator bradykinin, which is involved in blood pressure control [1]. Similarly, dipeptidyl peptidase IV (DPP-IV) has emerged as another important therapeutic target due to its role in glucose metabolism and cardiovascular function by degrading incretin hormones, such as glucagon-like peptide-1 (GLP-1) [2]. DPP-IV is widely expressed in the vascular system, including endothelial cells, macrophages, and cardiomyocytes, which in turn regulates inflammation and vascular function [3]. Both ACE and DPP-IV are involved in diseases related to metabolic syndrome, such as hypertension or diabetes, and are clinically targeted for treatment. However, clinical studies have revealed that synthetic inhibitors targeting ACE or DPP-IV show significant adverse effects. For example, ACE inhibitors commonly cause dry cough, hypotension, dizziness, and hyperkalemia and can lead to potentially life-threatening angioedema [4,5]. For DPP-IV inhibitors, upper respiratory tract infections, urinary tract infections, headaches, and nasopharyngitis have been reported as the common side effects [6,7]. Due to the aforementioned limitations, natural and safe bioactive peptides possessing both ACE- and DPP-IV-inhibitory properties have gained augmenting interest.

Due to the adverse impact of synthetic drugs, bioactive peptides have gained significant attention as natural therapeutic agents. Recently, peptides that can inhibit ACE and DPP-IV, which are involved in blood pressure regulation and glucose homeostasis, have been searched [8]. Bioactive peptides typically consist of 2–20 amino acid residues and are inactive within their parent proteins. Those active peptides can be released through enzymatic hydrolysis. The production of bioactive peptides is normally employed by using an enzymatic hydrolysis process with specific proteases, such as alcalase, Flavourzyme, or trypsin [9]. The hydrolysates from different proteins often contain a variety of peptides that can simultaneously inhibit both ACE and DPP-IV through competitive inhibition and/or enzyme–substrate binding interference [10]. In particular, a wide range of plant sources has been reported for isolation of ACE- and DPP-IV-inhibitory peptides. For example, wheat, peas, mushrooms (*Pleurotus cornucopiae*), soybeans, walnuts, date seed flour, bitter melon seeds, and spinach have been explored as promising substrates for ACE-inhibitory peptides [10]. For DPP-IV inhibition, chickpea (*Cicer arietinum*) proteins were enzymatically hydrolyzed, and peptides such as HIS-PHE were identified as strong DPP-IV inhibitors with favorable pharmacokinetic properties [11]. These examples highlight the diversity of plant-derived peptides that can serve as dual or specific inhibitors, supporting the increasing interest in plant proteins as functional ingredients for the management of hypertension and type 2 diabetes.

For a better understanding of the modes of action of peptides in inhibiting the target enzymes, some computational approaches, such as molecular docking, have been applied for the identification of these bioactive peptides through in silico analysis. This technique can simulate and examine the interactions between peptide and enzyme by modeling the three-dimensional structures and calculating binding energies at specific active sites. Therefore, key binding pockets of DPP-IV and ACE are commonly used to predict the inhibitory potential of peptides [11,12]. Additionally, machine learning algorithms, which have been trained on experimental data, can screen peptide sequences for their bioactivity based on physicochemical properties like hydrophobicity, charge, and size [12]. This integrated computational–experimental approach is significantly useful for the prediction of potential bioactive peptides derived from different protein sources.

Legume proteins have emerged as excellent sources for bioactive peptides due to their high protein content (20–35%) and variety in amino acid compositions [13,14]. Bambara groundnut (*Vigna subterranea* L. Verdc) is an underutilized African legume with remarkable potential for enhancing global food security because it grows in inappropriate environments [15]. The crop demonstrates exceptional drought tolerance and can grow in poor soils. It requires minimal agricultural inputs, which is currently suitable under situations where climate change and environmental degradation become dominant [16]. Commonly, Bambara groundnut has been cultivated in sub-Saharan Africa by small-scale farmers; however, it has also been successfully introduced and cultivated in Southeast Asia, particularly in Thailand, where it is known as “tua raung” [15,17]. Bambara groundnut without pericarp contains 17–25% protein, 55–70% carbohydrates, 5–7% fiber, and 6–12% lipids [17,18,19]. It also contains important minerals, such as iron, zinc, potassium, and calcium [18]. Despite these advantages, Bambara groundnut remains underexploited commercially, though it is traditionally consumed in various forms.

Although Bambara groundnut shows high protein content and favorable amino acid profile, no research on bioactive peptides from Bambara groundnut protein hydrolysates exists. Although the preliminary studies indicated the potential antioxidant activity in Bambara groundnut protein fractions [20], the peptides in protein hydrolysates exhibiting specific bioactivities have not been identified, particularly using modern analytical and bioinformatics approaches. Therefore, this study aimed to explore bioactive peptides with dual ACE- and DPP-IV-inhibitory properties from Bambara groundnut protein isolate through enzymatic hydrolysis. Bioinformatics and molecular docking approaches were also employed to analyze the interactions between the peptides and the target enzymes. Binding energies at specific active sites, enabling efficient interaction between peptides and enzymes, were also calculated.

## 2. Materials and Methods

### 2.1. Preparation of Protein Isolate from Bambara Groundnut

The protein isolate was prepared according to the method of Kudre et al. (2013) [21]. Bambara groundnut seeds were washed and dried in a hot air oven at 60 °C for 24 h. The dried seeds were dehulled, and the seed coat was removed from the seeds. The seeds were ground using a high-speed grinder for 30 s and then sieved through a 60-mesh screen. The protein isolate was prepared by dispersing the obtained powder in distilled water at a ratio of 1:10 (*w*/*v*). The pH of the mixture was adjusted to 12 using 2 M NaOH solution. The mixture was stirred at 150 rpm for 2 h at 28–30 °C. The mixture was subsequently centrifuged at 8000× *g* for 30 min at 25 °C. The clear protein solution was adjusted to pH 4.5 using 6 M HCl to precipitate the protein. The protein precipitate was collected by centrifugation (8000× *g*, 30 min) and washed with distilled water at a ratio of 1:10 (*w*/*v*) before centrifugation under the same conditions. The protein isolate was collected and freeze-dried at −50 °C for 72 h. Dried powder kept in a zip-lock bag was stored in a refrigerator, and the storage time was not longer than 2 months.

### 2.2. Preparation of Protein Hydrolysate

Protein isolate powder was dispersed in distilled water at 10% (*w*/*v*). The hydrolysates were prepared using Flavourzyme (≥500 U/g; Sigma-Aldrich, MO, USA) at 50 °C and pH 7.0. The enzyme was added to the dispersion at enzyme-to-substrate ratios of 2% and 4% (*w*/*v*). The mixtures were incubated for 15, 30, 60, 90, 120, 180, and 240 min. The digested samples were collected and heated at 95–100 °C for 10 min to terminate the enzyme before centrifugation (4000× *g*, 10 min). The supernatant was collected and tested for degree of hydrolysis (DH) using the TNBS (trinitrobenzenesulfonic acid) method [22].

### 2.3. Determination of α-Amino Group Content and Degree of Hydrolysis

The protein hydrolysate samples were mixed with 5% sodium dodecyl sulfate (SDS) solution at a ratio of 1:1 and incubated at 85 °C for 15 min to dissolve all peptides and undigested proteins. The content of the α-amino group was determined according to the method of Benjakul and Morrissey (1997) [22]. The sample solution was mixed with 2 mL of 0.2 M phosphate buffer, pH 8.2, and 1 mL of 0.01% (*w*/*v*) trinitrobenzenesulfonic acid (TNBS) solution. The mixture was incubated in the dark at 50 °C for 30 min before adding 2 mL of 0.1 M Na_2_SO_3_ solution. The absorbance was measured at 420 nm using FLUOstar^®^ Omega microplate reader (BMG Labtech, Ortenberg, Germany). The α-amino group content was computed from a standard curve of L-Leucine (0–2 mM) and expressed as the mmole equivalent of leucine/mL.

The degree of hydrolysis (DH) was determined using the following equation [22]:

DH (%) = [(L − L_0_)/(L_max_ − L_0_)] × 100

where L_0_ was the content of α-amino group of the initial sample dispersion, L was the content of α-amino group of the sample at various hydrolysis times, and L_max_ was the total α-amino group content of the sample hydrolyzed with 6 M HCl at 110 °C for 24 h.

### 2.4. Separation of Peptides Using Ultrafiltration

Bambara groundnut protein hydrolysate (100 mL) was fractionated using an Amicon ultrafiltration system equipped with membranes of varying molecular weight (MW) cut-offs to obtain peptide fractions of different sizes: >10 kDa, 5–10 kDa, 3–5 kDa, 1–3 kDa, and <1 kDa. Initially, the 100 mL hydrolysate was passed through a 10 kDa cut-off membrane in a vacuum until the retentate volume was reduced to approximately 20 mL. This retentate, containing peptides larger than 10 kDa, was collected and designated as the >10 kDa fraction. The filtrate from this first step, containing peptides smaller than 10 kDa, was subsequently subjected to sequential ultrafiltration using membranes with 5, 3, and 1 kDa cut-offs to obtain the corresponding 5–10 kDa, 3–5 kDa, 1–3 kDa, and <1 kDa fractions. Each fraction was collected, stored appropriately, and used for the determination of ACE- and DPP-IV-inhibitory activities. For enzyme inhibition assays, each peptide fraction was diluted with deionized water to final concentrations of 8 mg/mL and 6 mg/mL. The protein content of each fraction was determined using the Pierce™ Modified Lowry Protein Assay Kit (Thermo Fisher Scientific, MA, USA), following the manufacturer’s protocol.

### 2.5. Angiotensin-Converting Enzyme (ACE) Inhibition Assay

The ACE-inhibitory activity was measured using an ACE Kit-WST (Dojindo Laboratories, Japan) following the manufacturer’s instructions. Briefly, different fractions of protein hydrolysate were diluted properly with deionized water. For the assay, 20 μL of prepared fractions was added to a 96-well microplate. Thereafter, 20 μL of substrate solution and 20 μL of enzyme working solution were added. The mixture was incubated at 37 °C for 1 h. Subsequently, 200 μL of indicator working solution was added to each well and incubated at room temperature for 10 min. The absorbance was read at 450 nm using a microplate reader. ACE-inhibitory activity was calculated using the equation:

ACE-inhibitory activity (% inhibition) = [(A_blank 1_ − A_sample_)/(A_blank 1_ − A_blank 2_)] × 100

where blank 1 is the positive control (without ACE inhibitor) and blank 2 is a reagent blank.

### 2.6. Dipeptidyl Peptidase IV (DPP-IV) Inhibition Assay

DPP-IV-inhibitory activity was evaluated using a DPP-IV Inhibitor Screening Assay Kit (Cayman Chemical, MI, USA) according to the manufacturer’s instructions. Briefly, 30 μL of diluted assay buffer and 10 μL of diluted DPP-IV were added to sample wells of 96-well plate, followed by 10 μL of fractions. Background wells contained 40 μL of assay buffer and 10 μL of deionized water, while initial activity wells consisted of 30 μL of assay buffer, 10 μL of enzyme, and 10 μL of deionized water. The reaction was initiated by adding 50 μL of substrate solution (Gly-Pro-aminomethylcoumarin) to all the wells. The plate was incubated at 37 °C for 30 min, and fluorescence intensity was measured using an excitation wavelength of 350 nm and an emission wavelength of 450 nm. DPP-IV-inhibitory activity was calculated using the following equation:

% Inhibition = [(Initial Activity − Activity with sample)/(Initial Activity − Background)] × 100

### 2.7. Liquid Chromatography–Tandem Mass Spectrometry (LC-MS/MS) Analysis

LC-MS/MS analysis was performed using an EASY nano-LC 1000 system coupled to a Q-Exactive™ Plus Hybrid Quadrupole-Orbitrap™ Mass Spectrometer (Thermo Scientific, MA, USA). Each peptide fraction obtained from ultrafiltration (>10 kDa, 5–10 kDa, 3–5 kDa, 1–3 kDa, and <1 kDa) was separately analyzed. For each fraction, 100 µg of peptides was prepared for in-solution digestion. Samples were denatured in 8 M urea with 100 mM TEAB buffer containing 1× protease inhibitor cocktail. After protein quantification by the BCA assay, samples were reduced with 10 mM DTT at 37 °C for 30 min and alkylated with 10 mM iodoacetamide at room temperature for 30 min. The digested peptides were prepared using trypsin at an enzyme-to-substrate ratio of 1:50 (*w*/*w*) and incubated at 37 °C for 16 h. After digestion, peptides were desalted using Pierce™ C18 spin columns and concentrated prior to LC-MS/MS injection.

Peptides were separated on a 25 cm EASY-spray C18 column (75 µm internal diameter, Thermo Fisher Scientific, MA, USA) using a linear gradient of mobile phase A (0.1% formic acid in LC-MS grade water) and mobile phase B (0.1% formic acid in LC-MS grade acetonitrile) at a flow rate of 300 nL/min. The gradient was programmed as follows: 5% B at 0 min, 15% B at 60 min, 30% B at 70 min, 50% B at 80 min, and 98% B from 82 to 90 min. An electrospray voltage of 2.0 kV was applied. Full MS scans were acquired at 70,000 resolution with an AGC target of 3 × 10⁶, maximum injection time (IT) of 250 ms, and a scan range of 350–1400 *m*/*z*. MS/MS scans were performed at 17,500 resolution with an AGC target of 5 × 10^4^, maximum IT of 100 ms, an isolation window of 1.2 *m*/*z*, and a normalized collision energy (NCE) of 27.

### 2.8. Bioactive Peptide Databases and Computational Analysis

In silico analysis was performed by using the most abundant peptides (top 26%) with de novo scores >96 for all the fractions. Computational tools were employed for analysis by using PeptideRanker (http://distilldeep.ucd.ie/PeptideRanker/; accessed on 10 February 2025) and BIOPEP-UWM (https://biochemia.uwm.edu.pl/biopep/peptide_data.php; accessed on 17 February 2025). PeptideRanker was used to assess bioactivity potential using a 0–1.0 scoring system with a 0.5 threshold [23]. ACE- and DPP-IV-inhibitory potentials were assessed using BIOPEP-UWM: Bioactive peptides [24]. The top three highest ranks of predicted ACE- and DPP-IV-inhibitory peptides were further used for molecular docking.

### 2.9. Modeling of Peptide Structures and Molecular Docking

Peptide structures were predicted using the PEP-FOLD peptide structure prediction server (https://bioserv.rpbs.univ-paris-diderot.fr/services/PEP-FOLD3/; accessed on 5 March 2025). The server utilizes a de novo approach based on the integration of sequence-based modeling and molecular dynamics simulations with a coarse-grained force field to predict and refine the 3D structures of the peptides [25]. The predicted peptide models were subsequently validated by the Ramachandran plot using the PROCHECK implemented in the PDBsum web server (https://www.ebi.ac.uk/thornton-srv/databases/pdbsum/Generate.html; accessed on 7 March 2025).

The binding patterns between predicted peptides and ACE or DPP-IV were studied using molecular docking techniques with the ClusPro web server program. The three-dimensional structure of the human ACE enzyme was imported from the protein data bank (PDB) database with PDB code 1o8a, and the three-dimensional structure of the human DPP-IV enzyme was imported with PDB code 1nu6. The cluster with the highest number of members after prediction with ClusPro was used as the representative of the most likely binding pattern. The dissociation constant (Kd) and binding affinity value (ΔG) were then analyzed using the PRODIGY (PROtein binDIng enerGY prediction) program [26]. The amino acids of each binding pattern were analyzed using the pyDockEneRes server [27]. Three-dimensional structures were visualized using Visual Molecular Dynamics (VMD) 1.9.3 software [28].

### 2.10. Statistical Analysis

Completely randomized design (CRD) was used throughout the entire study. The experimental data were presented as mean  ±  standard deviation (SD). The statistical significance was analyzed using a parametric one-way ANOVA. Tukey’s Honestly Significant Difference (HSD) test was used for mean comparison. A *p* value < 0.05 was considered statistically significant. SPSS software 20.0 (SPSS Inc., Chicago, IL, USA) was used for all analyses.

## 3. Results

### 3.1. Degree of Hydrolysis and Inhibitory Activities of Bambara Groundnut Protein Hydrolysates

**Figure 1:**
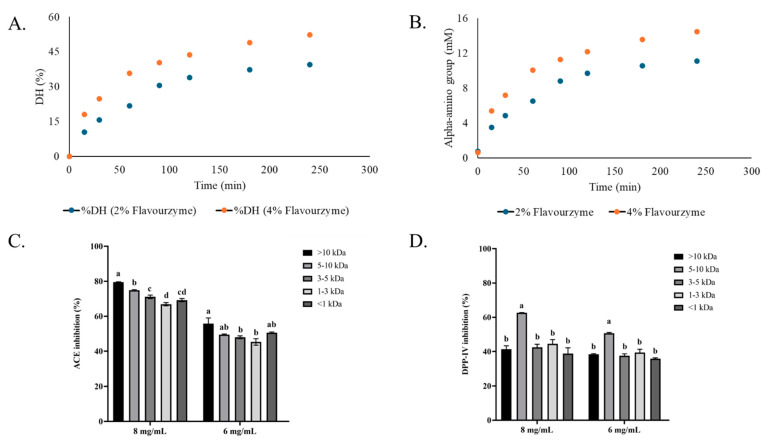
Degree of hydrolysis (**A**), α-amino group content (**B**) during hydrolysis with 2% and 4% Flavourzyme as a function of hydrolysis time, and ACE- (**C**) and DPP-IV- (**D**) inhibitory activities of fractions having different molecular weights (>10, 5–10, 3–5, 1–3, <1 kDa) at the concentrations of 8 and 6 mg/mL. Different lowercase letters on the bars within the same concentration of fraction denote significant differences (*p* < 0.05).

DH and α-amino group content of Bambara groundnut protein hydrolysates increased progressively with hydrolysis time for both Flavourzyme concentrations (2 and 4%) (Figure 1A,B). Notably, hydrolysis with 4% Flavourzyme yielded significantly higher DH and α-amino group content at the same hydrolysis time compared to that using 2%. The result indicated enhanced hydrolysis efficiency when higher enzyme concentrations were employed. The highest ACE-inhibitory activity was observed in the >10 kDa fraction across all concentrations (8 and 6 mg/mL). The inhibitory activity decreased when smaller peptide fractions were tested (Figure 1C). However, there was no significant difference between fraction <1 and 1–3 kDa fractions (*p* > 0.05). In contrast, DPP-IV-inhibitory activity was more pronounced for the 5–10 kDa fraction at 8 and 6 mg/mL. No differences in DPP-IV-inhibitory activity were found for the rest of the fractions, regardless of size distribution (Figure 1D). These findings suggest that Bambara groundnut hydrolysates, especially those derived from 4% Flavourzyme treatment, exhibit potent ACE- and DPP-IV-inhibitory activities. Nonetheless, the inhibition of both enzymes depended on peptides present in hydrolysate. Moreover, the peptide size had a significant influence on the inhibition of both enzymes.

### 3.2. In Silico Screening and Characteristics of Bioactive Peptides

A total of 191 peptides were identified from the hydrolysate from the Bambara groundnut protein isolate using Flavourzyme based on the top 26% abundance with *de novo* score >96% (Table 1). The highest number of these peptides was observed in the >10 kDa and 3–5 kDa fractions. Among them, those peptides with PeptideRanker scores >0.5 were predominantly found in the 5–10 kDa and >10 kDa fractions, suggesting a greater potential for bioactivity. Moreover, peptides with predicted ACE– and DPP-IV-inhibitory scores >0.5 were detected in all fractions, irrespective of MW ranges. However, the fraction with MW of 5–10 kDa contained the peptides with the highest DPP-IV-inhibitory activity. These findings indicate that medium-to-high-MW peptides of Bambara groundnut are more likely to serve as valuable sources of multifunctional bioactive peptides.

The peptides showing the highest activity prediction scores based on their predicted inhibitory potential using the BIOPEP database were selected from the identified pool (Table 2). For ACE inhibition, the three peptides with the highest BIOPEP scores, including peptides F3-1 (YKDGLYSPHW), F4-1 (LPVSTPGKF), and F5-6 (RPFLPPR), were selected. For DPP-IV inhibition, three peptides were found to share the same top prediction score (0.83), including F4-4 (EPWWPK), F4-6 (LLTPKF), and F5-3 (NLLMPH). These top peptides were then subjected to molecular docking analysis, and their binding affinity (ΔG) and dissociation constant (Kd) values were varied. ACE-inhibitory peptides exhibited strong interactions with ACE. ΔG values ranging from –10.2 to –11.3 kcal/mol and Kd values in the nanomolar range (4.0 × 10^−8^ to 1.1 × 10^−8^ M) were obtained. Similarly, DPP-IV-inhibitory peptides showed favorable docking results, with ΔG values from –8.6 to –9.1 kcal/mol and Kd values between 4.1 × 10^−7^ and 9.2 × 10^−7^ M. These results suggest that the selected peptides formed energetically favorable and stable complexes with the target enzymes.

### 3.3. Molecular Docking Interactions of Peptides with ACE

**Figure 2:**
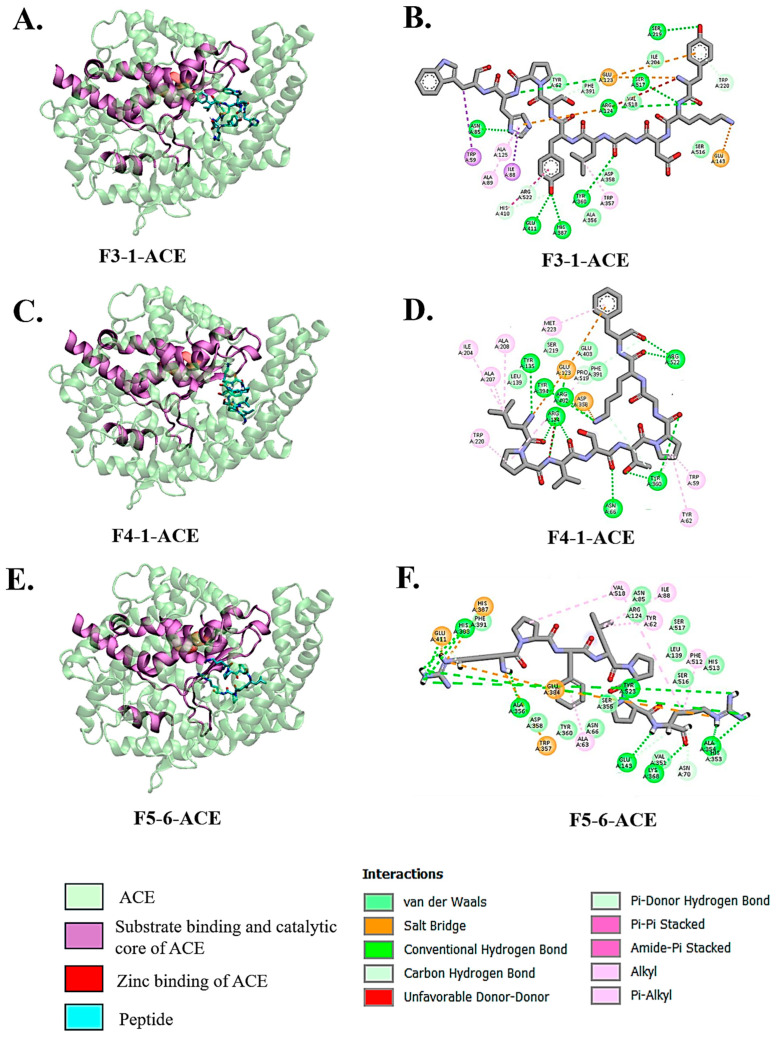
Molecular docking interactions of ACE-inhibitory peptides with ACE. (**A**,**B**) Peptide F3-1, (**C**,**D**) peptide F4-1, and (**E**,**F**) peptide F5-6 docked within the active site of human ACE. Left panels (**A**,**C**,**E**) show the overall binding orientation within the substrate-binding cleft and zinc-binding region. The right panels (**B**,**D**,**F**) illustrate detailed 2D interaction maps highlighting hydrogen bonds (green), alkyl/π–alkyl interactions (pink), salt bridges (orange), and van der Waals contacts (light green).

Molecular docking and visualization using Discovery Studio revealed that the selected peptides, F3-1 (YKDGLYSPHW), F4-1 (LPVSTPGKF), and F5-6 (RPFLPPR), interacted directly with the catalytic and zinc-binding regions of ACE (Figure 2A–F). Peptide F3-1 demonstrated an extensive interaction profile by forming hydrogen bonds with His387, Glu411, Arg124, Asn85, Ser517, Ser219, and Tyr360, as well as a salt bridge with Glu143 and Glu123 (Figure 2B). Additionally, the peptide had π–π, π–alkyl, and alkyl interactions with Trp59, Ile88, Ala125, Ala89, and Trp357. Peptide F4-1 was engaged in hydrogen bonding with Tyr135, Tyr394, Arg402, Arg124, Arg522, Tyr360, and Asn66 and formed a notable salt bridge with Glu358 and Glu123 (Figure 2D). The peptide’s interaction also included π–alkyl and alkyl contacts with residues such as Ile204, Ala207, Ala208, Met223, Trp220, Trp59, and Tyr62 contributing to stable positioning within the substrate-binding groove. Peptide F5-6 displayed hydrogen bonds with His383, Phe391, Ala356, Glu143, Lys368, Ala354, and Tyr523, and formed salt bridges with His387, Glu411, and Glu384 (Figure 2F). Additional π-alkyl and alkyl interactions with Val518, Ile88, Tyr62, Phe512, and Ala63 provided further stabilization of the complex. All peptides aligned well within the substrate-binding and catalytic core of ACE (Figure 2A,C,E), suggesting a mechanism of inhibition through direct competition at the active site.

### 3.4. Molecular Docking Interactions of Peptides with DPP-IV

**Figure 3:**
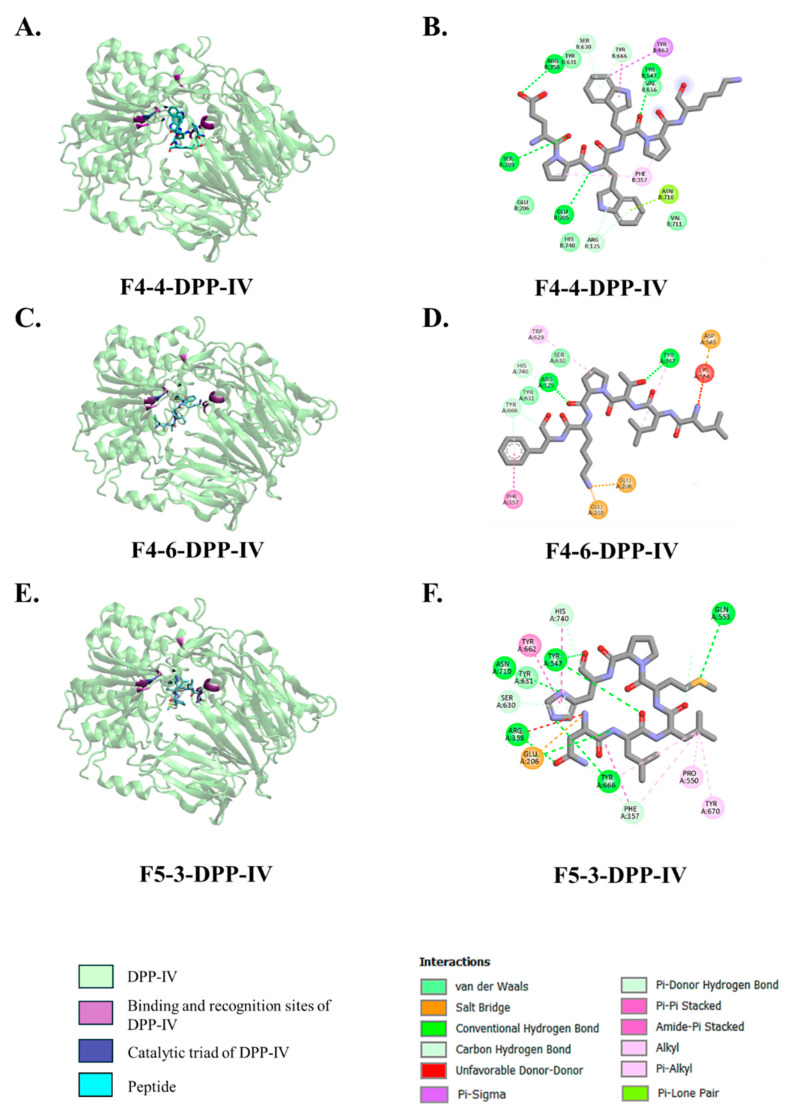
Molecular docking interactions of DPP-IV-inhibitory peptides with human DPP-IV. (**A**,**B**) Peptide F4-4 (EPWWPK), (**C**,**D**) peptide F4-6 (LLTPKF), and (**E**,**F**) peptide F5-3 (NLLMPH) docked with human DPP-IV. Panels (**A**,**C**,**E**) show the overall binding orientation of each peptide within the substrate-binding cavity. Panels (**B**,**D**,**F**) illustrate 2D interaction maps, highlighting various non-covalent interactions including conventional hydrogen bonds (green), salt bridges (orange), π–π and π–alkyl interactions (purple and light pink), and alkyl contacts (pink).

Molecular docking revealed that the top three predicted DPP-IV-inhibitory peptides, namely F4-4 (EPWWPK), F4-6 (LLTPKF), and F5-3 (NLLMPH), were positioned within the substrate-binding cavity of DPP-IV and interacted with residues surrounding the catalytic triad (Ser630, Asp708, and His740) as well as key binding pocket residues. The overall three-dimensional structures of the docking complexes were visualized using VMD, while detailed chemical bonding interactions, such as hydrogen bonds, salt bridges, and hydrophobic contacts, were analyzed and illustrated using Discovery Studio Visualizer (Figure 3A–F). These interactions involved hydrogen bonds, π-based interactions, alkyl contacts, and salt bridges. Peptide F4-4 (EPWWPK) formed hydrogen bonds with Glu205, Arg358, and Ser209, located near the entrance and lateral wall of the DPP-IV-binding pocket (Figure 3B). It also exhibited π–sigma and π–alkyl interactions with Tyr662 and Phe357, along with alkyl contacts involving Val207 and Pro550. Peptide F4-6 (LLTPKF) established hydrogen bonds with Arg125 and Tyr547 (Figure 3D) and showed π–π stacking with Phe357 and π–alkyl interactions with Tyr629. Three salt bridges were observed at Asp545, Glu206, and Glu205, and additional alkyl interactions were formed with Val207 and Val711. Peptide F5-3 (NLLMPH) formed hydrogen bonds with Asn710, Tyr547, Gln553, Arg358, and Tyr666 (Figure 3F). It was also engaged in π–π interactions with Tyr662, π–alkyl contacts with Pro550 and Tyr670, and alkyl interactions with Val207. A salt bridge was also identified at Glu206.

## 4. Discussion

This study demonstrated that protein hydrolysates derived from Bambara groundnut (*Vigna subterranea*) contained peptides with dual inhibitory activities against ACE and DPP-IV, suggesting the promising potential for managing hypertension and type 2 diabetes through natural dietary intervention. The enzymatic hydrolysis using Flavourzyme led to the generation of bioactive peptides, particularly those with MW >10 kDa and in the range of 5–10 kDa, which showed the highest inhibitory effects against ACE and DPP-IV, respectively. Although all peptide fractions exhibited ACE-inhibitory activities in the range of 70–80% at 8 mg/mL dose, no marked differences were observed among them, indicating that most peptides in the hydrolysate contributed similarly to ACE inhibition. This observation aligns with findings from other studies where fractionation did not significantly affect biological activities, suggesting that the crude hydrolysate retained substantial bioactivity [29]. In contrast, for DPP-IV inhibition, a significant difference was noted, with the 5–10 kDa fraction (fraction 4) showing the highest inhibitory activity (60% inhibition at 8 mg/mL dose) compared to other fractions (approximately 40% inhibition at 8 mg/mL dose). Thus, the peptides with particular chain lengths profoundly impacted their inhibition of DPP-IV. Overall, the use of hydrolysate in the crude form could simplify production processes and reduce costs through membrane fractionation, thereby enhancing its applicability in functional foods or nutraceuticals aimed at managing metabolic disorders.

The use of Flavourzyme for the hydrolysis of Bambara groundnut protein isolate is particularly interesting, as this enzyme has been widely employed to prepare bioactive peptides with diverse biological activities [30,31]. Flavourzyme possesses both endopeptidase and exopeptidase activities, enabling broad cleavage of peptide bonds in protein substrates and resulting in the release of peptides with varying lengths and sequences [30]. Moreover, it also contains exopeptidase, which can remove the amino acids from N- or C-termini. The broad specificity not only contributes to a higher degree of hydrolysis but also enhances the probability of releasing peptide sequences with bioactive motifs. Flavourzyme-treated hydrolysates often contain multifunctional peptides with antioxidant, antihypertensive, and anti-diabetic properties [31]. Importantly, in contrast to other proteases such as alcalase, which tend to generate bitter-tasting peptides, Flavourzyme is recognized for producing hydrolysates with more favorable organoleptic properties due to its exopeptidase activity, which can remove bitter hydrophobic residues from peptide termini [32]. This feature enhances the potential application of Bambara groundnut hydrolysates in functional foods, where both bioactivity and consumer acceptability are critical.

The integration of bioinformatics tools such as PeptideRanker and BIOPEP-UWM has emerged as a powerful approach in predicting bioactive peptides from protein hydrolysates [33,34,35]. In the present study, PeptideRanker was employed to assess the probability of bioactivity based on peptide sequence features, while BIOPEP-UWM enabled the functional annotation of peptides by matching them against the known bioactive motifs [23,24]. Numerous recent studies have validated the utility of this computational strategy for identifying ACE- and DPP-IV-inhibitory peptides. For example, Purcell et al. (2023) used PeptideRanker and BIOPEP to screen hydrolysates of oarweed (*Laminaria digitata*) and identified peptides such as IGNNPAKGGLF and YIGNNPAKGGLF with strong ACE-inhibitory activity [35]. Similarly, BIOPEP-UWM was used to perform in silico enzymatic hydrolysis of milkfish (*Chanos chanos*) to predict peptides with known ACE- and DPP-IV-inhibitory motifs, while PeptideRanker was applied to score and prioritize novel peptides based on predicted bioactivity [36]. These examples collectively demonstrate the robustness and reproducibility of bioinformatics-driven peptide screening, especially when enzymatic hydrolysis and proteomics data were linked.

Molecular docking analysis of Bambara groundnut-derived peptides provided compelling evidence for their inhibitory potential against ACE and DPP-IV. The top three peptides were predicted to inhibit ACE. These included F3-1 (YKDGLYSPHW), F4-1 (LPVSTPGKF), and F5-6 (RPFLPPR), which were able to interact directly with critical residues located within the enzyme’s active site and substrate-binding regions. The binding free energy (ΔG) of these ACE-inhibitory peptides ranged from –10.2 to –11.3 kcal/mol, with nanomolar dissociation constants comparable to classical ACE inhibitors such as lisinopril and RXP407 [37]. Peptide F3-1 showed extensive hydrogen bonding with His387 and Glu411, which are essential for zinc ion coordination in ACE catalysis [38,39,40]. It also formed salt bridges with Glu123 and Glu143, and hydrophobic interactions with Trp59, Ile88, and Trp357, indicating stable engagement with the enzyme’s S1 and S2 pockets. Peptide F4-1 interacted with Tyr135, Arg402, Asn66, and Tyr360, and formed salt bridges with Glu123 and Glu358, implying anchoring in both catalytic and allosteric regions. F5-6 interacted with His383 and Tyr523 via hydrogen bonds, closely aligning with interaction patterns observed for the Ang II, which functions as a competitive inhibitor of the C-domain of somatic ACE (sACE) [38]. Ang II generally binds in two different sliding conformations within the C-domain active site, stabilized by residues such as His383, His387, Tyr523, and Ala356 without undergoing further cleavage. These residues are also involved in interactions with the Bambara groundnut-derived peptides.

In addition, molecular docking analysis also revealed the inhibitory mechanism of peptides involving F4-4 (EPWWPK), F4-6 (LLTPKF), and F5-3 (NLLMPH) on the DPP-IV enzyme. Most identified peptides bind effectively within the enzyme’s active site, engaging directly with the catalytic triad (Ser630, Asp708, and His740) and the key residues lining in S1 and S2 pockets, including Glu205, Glu206, Arg125, Phe357, Arg358, Tyr547, Tyr662, and Tyr666 [41]. These interactions are important for inhibitory activity, as they mimic the enzyme’s natural substrates and prevent substrate access. For example, peptide F4-4 established hydrogen bonds with Glu205, Arg358, and Ser209, while engaging in π–π interactions with Tyr662 and Phe357, which are the key residues forming the lateral wall of the binding pocket. Similarly, peptide F4-6 formed strong hydrogen bonds with Arg125 and Tyr547, π–π stacking with Phe357, and salt bridges with Glu205, Glu206, and Asp545. All these residues are responsible for stabilizing the negative charge developed during catalysis [42]. Peptide F5-3 interacted with Asn710, Gln553, Tyr547, Arg358, and Tyr666 in pockets S1 and S2, forming a network via hydrogen and π–π interactions that likely interfered with the oxyanion hole and transition state stabilization [41,42,43,44]. Moreover, these natural peptides demonstrated a mode of inhibition comparable to that of conventional DPP-IV inhibitors like sitagliptin, which also target the catalytic and binding pocket residues [45]. However, peptides might offer unique advantages due to their structural flexibility, allowing them to establish a broader network through non-covalent interactions, including hydrogen bonds, hydrophobic contacts, and aromatic stacking, without enzymatic cleavage.

## 5. Conclusions

Bambara groundnut protein hydrolysates prepared using Flavourzyme are a rich source of multifunctional bioactive peptides with dual inhibitory activity against ACE and DPP-IV. The in silico prediction and molecular docking enabled the identification of several peptides, which showed strong binding affinities to catalytic and substrate-binding residues of both enzymes. These peptides exhibited interaction patterns analogous to those of conventional inhibitors; however, they provided advantages because of their natural originality and potential safety. These peptides’ structural compatibility and binding stability suggested a competitive inhibition mechanism similar to that of conventional synthetic inhibitors. These findings contribute to the growing field of food-derived bioactive peptides and support the development of functional foods or nutraceuticals aimed at managing hypertension and type 2 diabetes through natural and safe therapeutic alternatives.

## Figures and Tables

**Table 1:** Number of peptides found in different fractions of protein hydrolysate from Bambara groundnut prepared using Flavourzyme.

Criteria	Peptide Size (kDa)				
	<1	1–2	3–5	5–10	>10
Top 26% abundance with de novo score >96%	22	29	46	44	50
Peptide Ranker score >0.5	5	5	4	10	9
ACE-inhibitory peptide score >0.5	1	1	1	1	1
DPP-IV-inhibitory peptide score >0.5	4	3	2	9	6

**Table 2:** Top three peptides with the highest predicted ACE- and DPP-IV-inhibitory activities from Bambara groundnut protein hydrolysate.

Inhibitory Activities	Pep no f *	Peptide	Denovo Score	*m*/*z*	Chemical Formula	Bioranker Score	Activity Prediction Score	ΔG (kcal mol^−1^)	Kd (M)
ACE	F3-1	YKDGLYSPHW	98	633.30	C61H80N14O16	0.59	0.70	−10.5	4.0 × 10^−8^
	F4-1	LPVSTPGKF	98	473.27	C45H72N10O12	0.65	0.67	−10.2	6.3 × 10^−8^
	F5-6	RPFLPPR	96	441.76	C42H67N13O8	0.93	0.71	−11.3	1.1 × 10^−8^
DPP-IV	F4-4	EPWWPK	98	421.71	C43H55N9O9	0.92	0.83	−8.7	7.1 × 10^−7^
	F4-6	LLTPKF	97	359.72	C36H59N7O8	0.60	0.83	−9.0	4.6 × 10^−7^
	F5-3	NLLMPH	97	362.69	C32H53N9O8S	0.58	0.83	−9.1	4.1 × 10^−7^

* F refers to the fraction number (e.g., F3 = 3–5 kDa fraction, F4 = 5–10 kDa fraction), and the number after the dash refers to the peptide number within that fraction. ΔG: Gibbs free energy change from molecular docking; *m*/*z*: mass-to-charge ratio of the peptide ion; Kd: dissociation constant calculated from molecular docking.

## Data Availability

The original contributions presented in the study are included in the article. Further inquiries can be directed to the corresponding author.
